# Supplementary material for: Transcriptomic profiling of the middle temporal gyrus reveals differential glial/neuronal dysregulation across Alzheimer’s disease and aging
Source: bioRxiv. 2025 Oct 20:2025.10.19.683343. Preprint. [Version 1] doi: 10.1101/2025.10.19.683343 (PMC12633431; doi:10.1101/2025.10.19.683343)

***Figure S1****. Total sequenced reads distribution (in million) in the 621 sequenced samples.*


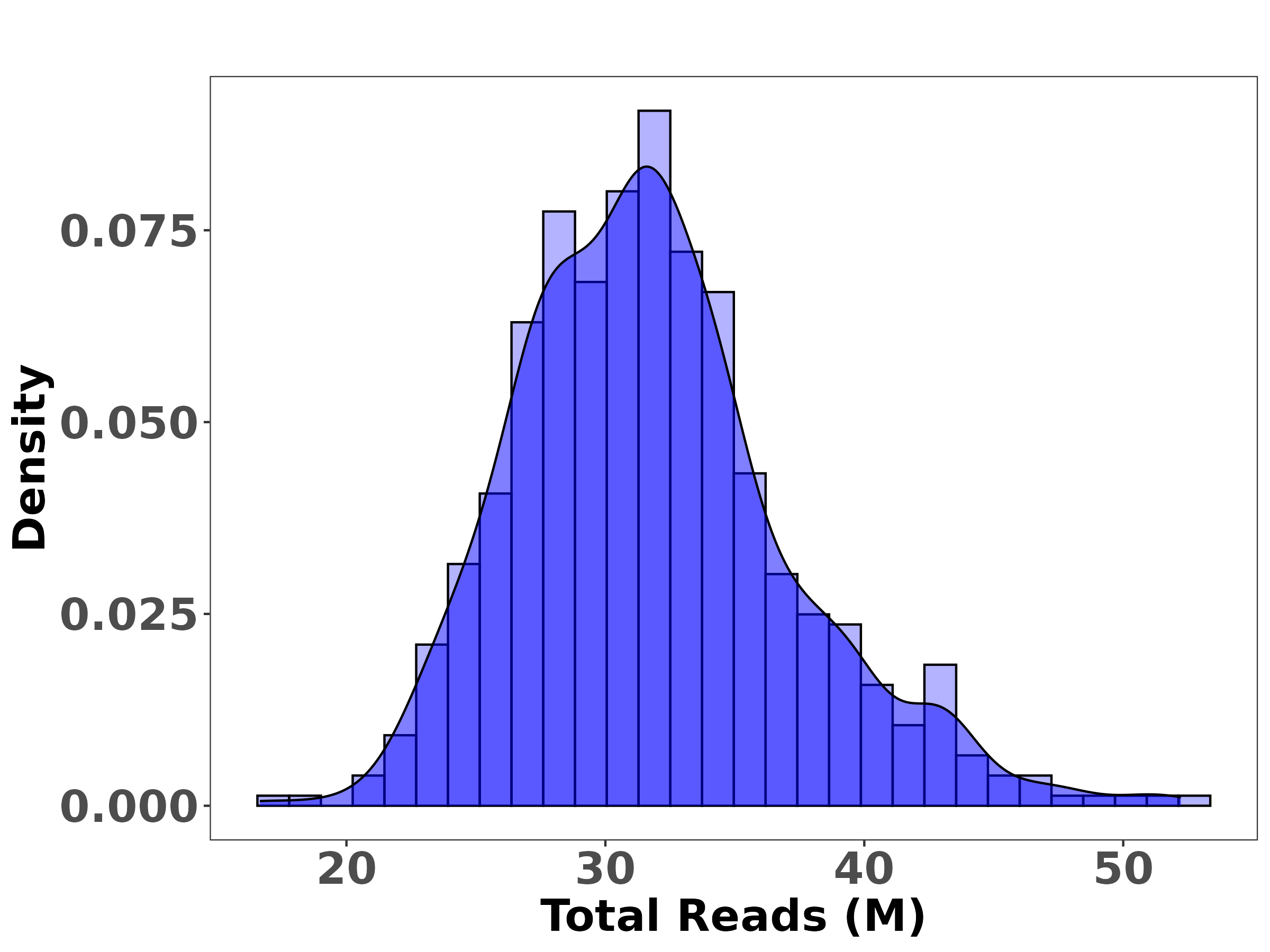


***Figures S2****. Uniquely mapped reads distribution as estimated by STAR in the 621 sequenced samples.*

***
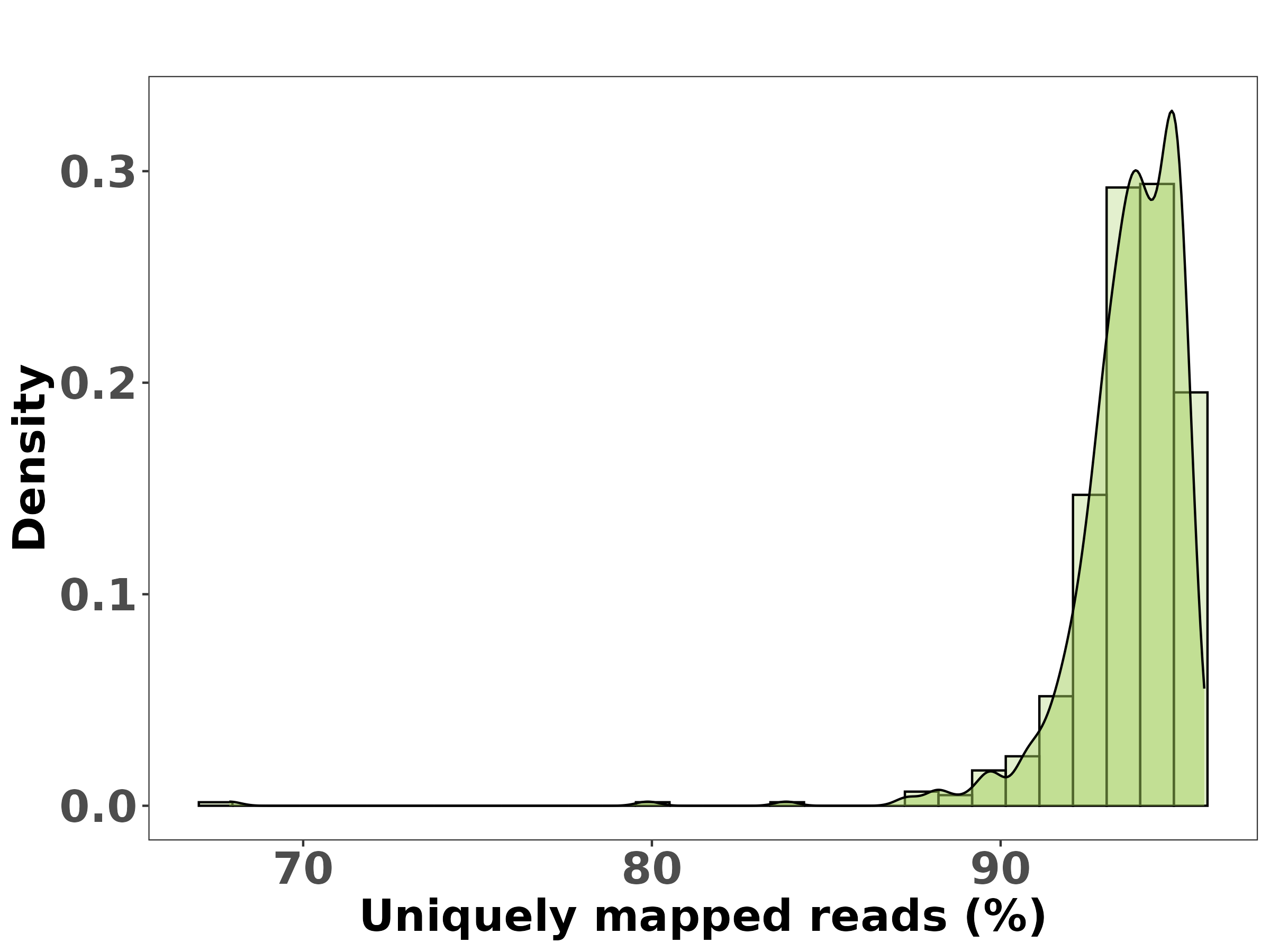
***

***Fig. S3.*** *Plot of the two top principal components. Red points are the 11 identified outliers (± 3 standard deviations across the mean of one of the two top components).*


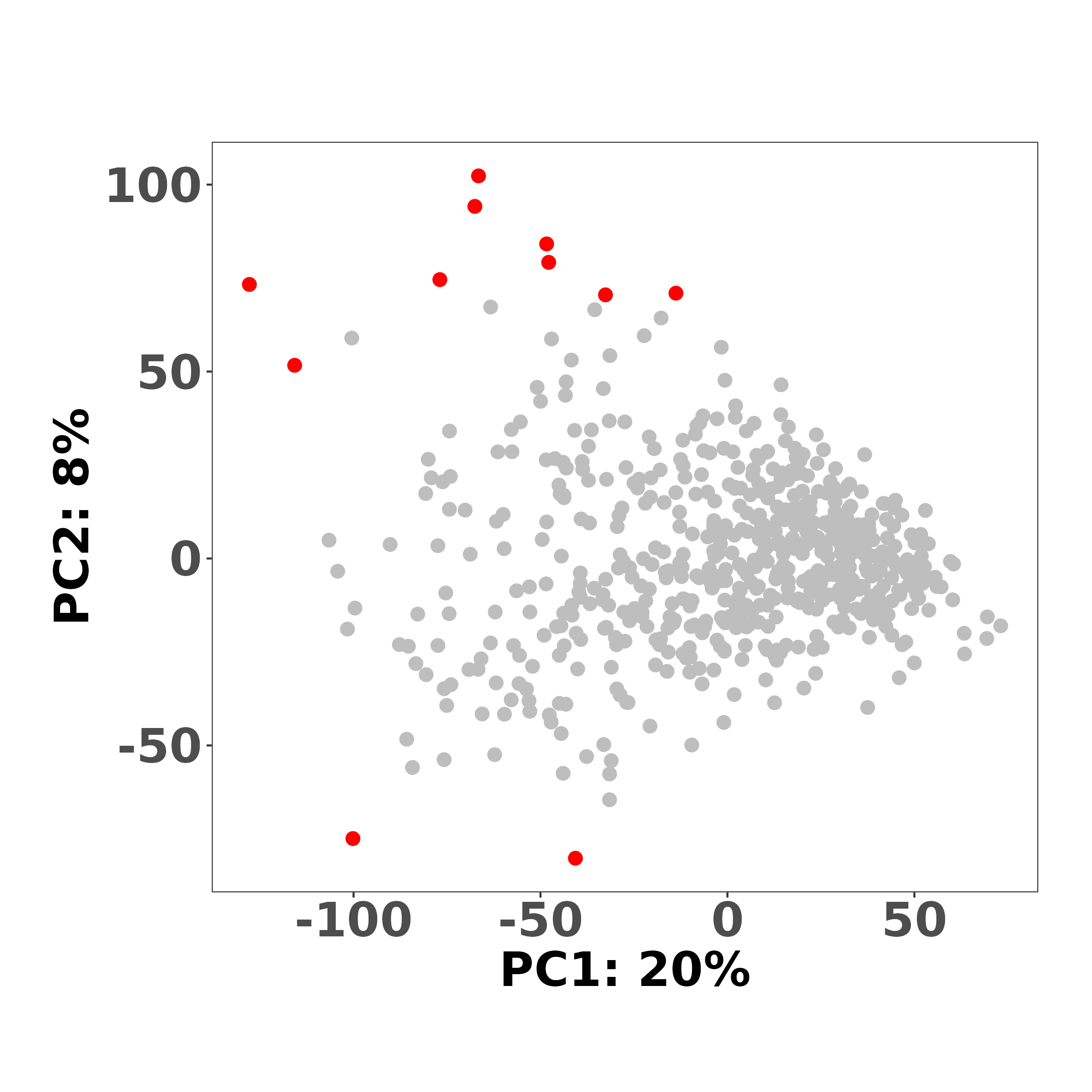


***Fig. S4.*** *Plot of the two top principal components after removing the 11 outliers (final sample size: n = 606).*


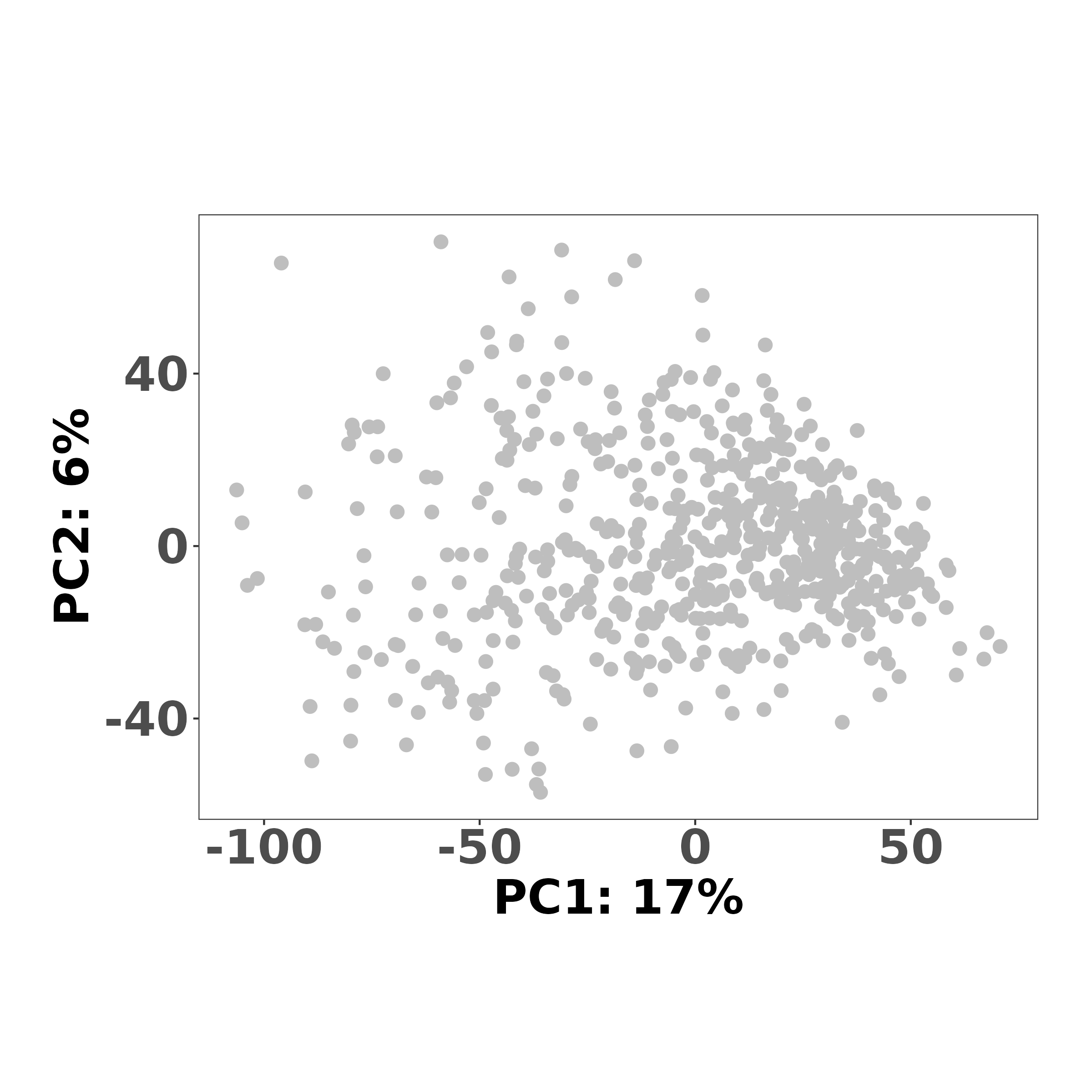

Supplement: Supplement 1 [file media-1.docx]
